# Challenges of driving CD30-directed CAR-T cells to the clinic

**DOI:** 10.1186/s12885-019-5415-9

**Published:** 2019-03-06

**Authors:** Natalie S. Grover, Barbara Savoldo

**Affiliations:** Lineberger Comprehensive Cancer Center, University of North Carolina, Chapel Hill, NC 27599 USA

**Keywords:** Cellular immunotherapy, Chimeric antigen receptor T cells, CD30, Hodgkin lymphoma, Anaplastic large cell lymphoma

## Abstract

Chimeric antigen receptor T (CAR-T) cells are a promising new treatment for patients with relapsed or refractory hematologic malignancies, including lymphoma. Given the success of CAR-T cells directed against CD19, new targets are being developed and tested, since not all lymphomas express CD19. CD30 is promising target as it is universally expressed in virtually all classical Hodgkin lymphomas, anaplastic large cell lymphomas, and in a proportion of other lymphoma types, including cutaneous T cell lymphomas and diffuse large B cell lymphomas. Preclinical studies with CD30-directed CAR-T cells support the feasibility of this approach. Recently, two clinical trials of CD30-directed CAR-T cells in relapsed/refractory CD30+ lymphomas, including Hodgkin lymphoma, have been reported with minimal toxicities noted and preliminary efficacy seen in a proportion of patients. However, improving the persistence and expansion of CAR-T cells is key to further enhancing the efficacy of this treatment approach. Future directions include optimizing the lymphodepletion regimen, enhancing migration to the tumor site, and combination with other immune regulators. Several ongoing and upcoming clinical trials of CD30-directed CAR-T cells are expected to further enhance this approach to treat patients with relapsed and refractory CD30+ lymphomas.

## Background

Chimeric antigen receptor T (CAR-T) cells have shown remarkable activity in hematologic malignancies. CAR molecules are created by fusing the single chain variable fragment (scFv) derived from an antibody targeting a surface antigen with the T cell signaling domain [1, 2]. These molecules are grafted on T cells though an engineering process that more commonly uses retro- or lentiviruses, or, in some instances, plasmids. CD19 is a rational target for B-cell malignancies, including lymphomas, as it is expressed on B cells during all stages of differentiation as well as in cells that have malignantly transformed [3]. CAR-T cells directed against CD19 have shown excellent responses in patients with relapsed or refractory B-cell lymphomas, particularly diffuse large B-cell lymphoma (DLBCL) with some durable remissions seen [4, 5], earning FDA approval for this indication. However alternative targets are needed for other types of lymphoma that lack CD19 expression, including diseases like classical Hodgkin lymphoma (HL), anaplastic large cell lymphoma (ALCL) and other T-cell lymphomas.

Although the majority of patients with HL are cured with first line therapies, about 15% of patients either have primary refractory disease or later relapse after an initial response to treatment [6]. The standard of care for patients who relapse after first line treatment is high dose chemotherapy followed by autologous stem cell transplant (ASCT) with about half of patients relapsing after transplant [7]. Unfortunately, the prognosis for these patients is poor, with allogeneic stem cell transplant (alloSCT) traditionally offering the best chance for sustained remission [8]. However, this treatment is also associated with significant morbidity and mortality. Novel therapies are needed for patients with relapsed and refractory HL.

ALCL is a subtype of peripheral T cell lymphoma with heterogeneous characteristics [9]. While the prognosis for anaplastic lymphoma kinase-positive (ALK+) ALCL is good, with 5 year survival rates generally ranging from 70 to 90%, ALK-negative ALCL has a more guarded prognosis, with 5 year survival rates of 40–60% [9]. Salvage therapy for patients with chemosensitive disease generally consists of high dose chemotherapy followed by ASCT. However, the prognosis for patients with relapsed/refractory ALCL who are not eligible for transplant or fail second-line therapy is dismal, with one retrospective study showing a median progression free survival and overall survival of 3 and 1.8 months, respectively [9]. Alternative treatment approaches are therefore needed for the treatment of patients with relapsed or refractory ALCL.

A defining characteristic for both HL and ALCL is the presence of a common surface molecule, CD30, a transmembrane receptor and member of the tumor necrosis factor (TNF) receptor superfamily [10, 11]. CD30 is also expressed in other lymphomas, including universally in lymphomatoid papulosis and in some cases of DLBCL, primary mediastinal B-cell lymphoma, mycoses fungoides, peripheral T cell lymphoma, and adult T-cell leukemia/lymphoma [12–14]. Novel treatments are also needed for these lymphomas, especially for patients who do not respond to initial treatment.

CD30 is an excellent candidate for immune-based therapies due to its restricted expression on tumor cells, with limited expression on a small subsets of activated normal (non-malignant) lymphocytes [15], leading to low risk for off tumor on target toxicity.

CD30 has been explored extensively as antibody based therapy, from naked to immuno-conjugated. The most remarkable results have been achieved with brentuximab vedotin (BV), an antibody drug conjugate directed against CD30, which has indeed shown good tolerance as well as promising activity in CD30+ lymphomas, with an overall response rate (ORR) of 75% and complete response (CR) rate of 34% in patients with relapsed or refractory HL [16] and an ORR of 86% and CR rate of 57% in relapsed or refractory systemic ALCL [17]. Although BV appears to have excellent responses, these are not usually durable with only 22% of patients with relapsed or refractory HL not having progressed after 5 years [18]. To overcome some of the challenges with antibody-based therapy, namely limited persistence [19, 20] and tumor penetration [20, 21], CAR-T cells have been explored. The success and tolerability of BV provided evidence towards the feasibility of targeting CD30 [16, 18] with CAR-T cells.

### Preclinical studies of CD30-directed CAR-T cells

The first studies of CAR-T cells targeting CD30 were performed in the late 1990’s by Hombach et al. and showed effective cytolysis of CD30+ HL cell lines in vitro [15, 22]. However these CAR molecules lacked co-stimulatory signaling, which limited their effectiveness. Savoldo et al. proposed to express this CAR molecule on virus (Epstein Barr Virus)-specific cytotoxic T cells (EBV-CTLs) to ensure that these chimeric cells received appropriate costimulatory signals over time. As expected, these cells maintained their ability to recognize and kill EBV+ tumors while, at the same time, targeting CD30+ cancer cells (such as HL and ALCL tumor cell lines) both in vitro and in vivo in a xenogeneic mouse model [23]. Subsequent advances in the engineering process have introduced co-stimulatory endodomains within the CAR molecule, which made manufacturing less cumbersome and the need for T-cells with dual antigen specificity obsolete [24, 25].

Regardless, these studies addressed major theoretical challenges associated with the targeting of the CD30 molecule. First of all, increased levels of soluble CD30 are present in the plasma of patients with HL and ALCL, raising concerns of competition for binding to the CAR [26]. However, in vitro studies demonstrated that elevated levels of soluble CD30 did not negatively impact the activity of CD30-directed CAR-T cells [15, 27], likely because the epitope targeted by the CAR is not retained in the soluble form of the molecule or because multiple immobilized molecules are required to activate CAR signaling.

Second, these studies explored expression levels for CD30 molecule to sensitize CAR-T cells killing. CD30 is expressed transiently by a subset of lymphocytes upon activation raising concerns for premature elimination of T or B cells during virus responses. Extensive ex vivo studies have however ruled out this issue [23], suggesting that the level of CD30 upregulation in memory T cells responding to virus-associated antigen stimulation is lower than that present on tumor cells, and thus unable to fully activate the killing machinery. Antigen sensing by CAR molecules is becoming an important characteristic, as differential expression of targetable molecules between normal vs tumor cells ultimately will dictate the selection of the scFv for applications of CAR against solid tumors. However, the potential for CD30-directed CAR-T cells to eliminate allo-reactive T cells or regulatory T cells (Tregs), which appear to express CD30 at a significantly higher level, remains to be fully explored, and may open this approach for other applications in the stem cells transplant field.

A final important aspect addressed in these preclinical studies dealt with the resistance of some CD30+ cells to CAR-T cell mediated killing. CD30 is indeed expressed by hematopoietic stem and progenitor cells (HSPCs) during activation [27, 28], potentially leading to disorders of hematopoiesis including bone marrow aplasia. However, when comparing the potency of CD30-directed CAR-T cells against CD30+ HSPCs and CD30+ MyLa cutaneous T cell lymphoma cells [27], minimal activity was observed against the former. Furthermore, HSPCs that were sorted into CD30+ and CD30– cells showed only slightly higher cytolysis in the presence of CAR-T cells which was nevertheless much lower compared to the lysis of MyLa lymphoma cells [27, 29]. HSPCs that were co-cultured with CD30-directed CAR-T cells also had normal myeloid colony formation, with only a slight decrease in erythroid colony formation. Importantly, the adoptive transfer of autologous CD30-directed CAR-T cells during HSPC reconstitution in humanized mice produced no impairment in human peripheral T and B cells, suggesting preserved hematopoiesis [27], and confirming lack of significant bone marrow toxicity.

In addition to the differential expression of CD30 on HSPCs at a level that is below the threshold for CAR-T cell activation, some intrinsic resistance of progenitor cells seems likely. HSPCs express higher levels of SP6/PI-9 serine protease which inactivates granzyme B, a major facilitator of T-cell mediated apoptosis [27, 29]. Although different cells use different strategies, this observation is consistent with studies in embryonic cells and tumors which, despite expressing CD30, are more resistant to CAR-T cell killing [30].

### Clinical trials of CD30-directed CAR-T cells

Two trials of CD30 directed CAR-T cells have been published to date, with both trials showing this treatment to be well tolerated with some anti-tumor activity (Table 1). The two studies utilized different scFv, costimulatory signals, delivery systems, preparation regimens and doses, making comparisons difficult to perform, while at the same time providing broad scenarios of function.

**Table 1:** Completed CD30-Directed CAR-T Cell Clinical Trials for Patients with Relapsed/Refractory CD30+ Lymphoma

Trial	Wang et al.	Ramos et al.
Costimulatory Domain	4-1BB	CD28
Viral Vector	Lentivirus	Gammaretrovirus
Conditioning Chemotherapy	FC, GMC, PC	None
Doses	1.1–2.1 × 10^7^ CAR-T cells/kg	2 × 10^7^, 1 × 10^8^, 2 × 10^8^ CAR-T/m^2^
Number of Patients Treated	18 patients (17 HL, 1 cutaneous ALCL)	9 patients (6 HL, 1 cutaneous ALCL, 1 systemic ALCL, 1 DLBCL evolved to HL)
Responses	ORR 39% (7 PR); 6 with SD	ORR 33% (2 CR, 1 continued CR); 3 with SD

*FC* fludarabine and cyclophosphamide, *GMC* gemcitabine, mustargen, cyclophosphamide, *PC* nab-paclictaxel and cyclophosphamide, *HL* Hodgkin lymphoma, *ALCL* anaplastic large cell lymphoma, *DLBCL* diffuse large B-cell lymphoma, *ORR* overall response rate, *PR* partial response, *SD* stable disease, *CR* complete response

Wang et al. treated 18 patients with relapsed/refractory CD30+ lymphoma (17 with HL and 1 with cutaneous ALCL) with an anti-CD30 CAR [31]. This CAR (derived from AJ878606.1 antibody) utilized the 4-1BB costimulatory endodomain and a lentiviral vector for T cell engineering. Out of the 18 patients treated, 9 had received prior ASCT and 5 had been treated with BV. Patients received a mean dose of 1.56 × 10^7^ CAR-T cells/kg after a lymphodepleting regimen, consisting of 3 different combinations, which caused some degree of cytopenias [31]. All of the patients had a grade 1 or 2 febrile infusion reaction (fevers and chills) that recovered overnight. There were only two grade 3 or higher toxicities: one patient had abnormalities in liver function tests felt to be secondary to toxicity from lymphodepletion and one patient had systolic dysfunction, likely related to prior anthracycline exposure. There was no cytokine release syndrome.

Out of 18 patients treated and evaluable for response, 7 patients had a partial response (PR) and 6 patients had stable disease (SD) after infusion There were no CR and the ORR was 39%. The median progression free survival was 6 months with 4 patients having continued response at time of publication. There were 5 patients who received a second CAR-T cell infusion, with 3 patients maintaining PR after 2nd treatment, 1 patient maintaining SD, and 1 patient obtaining a PR after being assessed as having SD after 1st infusion. Lymph nodes seemed to respond better to treatment than extranodal disease, and lung lesions appeared to respond the least to treatment, although it is difficult to make conclusions with such a small sample size.

In most patients treated, CAR transgene levels in the peripheral blood peaked at 3–9 days after infusion and decreased to baseline at 4–8 weeks after infusion Higher numbers of CAR transgenes as well as a decreased number of CD30+ tumor cells were found in the few patients who had tumor biopsies performed at that time, suggesting that functional CAR-T cells trafficked to tumor sites.

Ramos et al. reported the results of 9 patients with relapsed/refractory CD30+ lymphoma (6 with HL, 1 with cutaneous ALK negative ALCL, 1 with systemic ALK+ ALCL, and 1 with DLBCL evolved to HL) [32]. For this trial, the CAR CD30 (derived from the HSR3 antibody) was combined with a CD28 costimulatory endodomain and delivered into T cells via a gammaretroviral vector [32]. Out of the 9 patients treated, 8 had active disease at time of cell infusion. All patients were heavily pre-treated and had relapsed after 3 or more prior lines of therapy, 7 had been previously treated with BV, and 6 had relapsed after ASCT.

Patients received up to 2 × 10^8^ CD30-directed CAR-T cells/m^2^ with no lymphodepleting regimen administered prior to infusion [32]. The treatment was well tolerated with no attributable toxicities to CAR-T cells or episodes of cytokine release syndrome reported. The authors also monitored T cell immunity to viral antigens before and after infusion and found no difference in T cell response to common viral pathogens [32]. In addition, there were no reports of viral infections after treatment with CD30 CAR-T cells.

Out of 8 patients treated who had active disease at time of infusion, 2 patients went into CR with 1 patient with ALK+ ALCL maintaining CR for 9 months before relapse, and the other patient with HL continuing to be in CR for greater than 2.5 years at time of publication [32]. Three patients had SD and 3 patients had progressive disease. The one patient treated who was already in CR at time of infusion after receiving salvage chemotherapy post ASCT has maintained a CR for over 2 years at time of publication. Most responses were seen in patients who received the highest dose level. There was a dose-dependent expansion of CAR-T cells in peripheral blood and levels peaked within 1 week of infusion and declined afterwards, but CAR signals were still detectable 6 months after infusion in 6 patients [32].

Despite both studies demonstrating good tolerability and some effects, results are modest compared to those achieved with CD19-directed CAR-T cells. There are currently several ongoing clinical trials with different CD30 CAR-T cell constructs in relapsed/refractory lymphomas addressing ways to improve outcome (Table 2).

**Table 2:** Current CD30-Directed CAR-T Cell Clinical Trials for Relapsed/Refractory CD30+ Lymphoma

Clinicaltrials.gov Identifier	Conditioning Regimen	Doses	Included Ages	Estimated Enrollment	Location
NCT02259556^a^	Cy/flu	Not specified (dose escalation)	16–80 years	30	Chinese PLA General Hospital
NCT02690545	Flu/benda	1 × 10^8^ cells/m^2^, 2 × 10^8^ cells/m^2^	3 years and older	34	University of North Carolina
NCT02917083	Cy/flu (if post ASCT, T-cell infusion at least 14 days after transplant)	2 × 10^7^ cells/m^2^, 1 × 10^8^ cells/m^2^, 2 × 10^8^ cells/m^2^	12–75 years	18	Baylor College of Medicine
NCT03049449	Cy/flu	0.3 × 10^6^ cells/kg up to maximum dose of 18 × 10^6^ cells/kg	18–73 years	76	National Cancer Institute
NCT03383965	Not specified	Not specified (dose escalation)	2–80 years	20	Weifang People’s Hospital
NCT02663297	ASCT	2 × 10^7^ cells/m^2^, 1 × 10^8^ cells/m^2^, 2 × 10^8^ cells/m^2^	3 years and older	18	University of North Carolina
NCT02958410	Not specified	Not specified (dose escalation)	14 to 75 years	45	Southwest Hospital, China

*CAR-T* chimeric antigen receptor T-cell, *Cy* cyclophosphamide, *flu* fludarabine, *benda* bendamustine, *ASCT* autologous stem cell transplant

^a^This study allows enrollment of newly diagnosed patient who are unable to receive or complete standard chemotherapy

### Future directions of CD30-directed CAR-T cells

Dissecting strategies to enhance CD30-CAR T cells need to be stepwise and multifaceted.

**Fig. 1:**
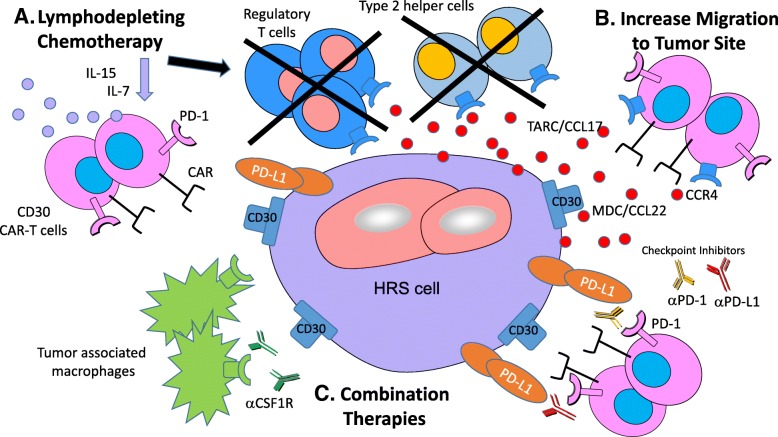
**a**. Lymphodepleting chemotherapy reduces the number of suppressive cells, such as regulatory T cells and type 2 helper cells, which can disturb the tumor microenvironment. It also stimulates production of cytokines, such as IL-7 and IL-15, which can promote expansion of CAR-T cells. **b**. Hodgkin Reed-Sternberg cells produce thymus and activation-regulated chemokine/CC chemokine ligand 17 (TARC/CCL17) and macrophage-derived chemokine (MDC/CCL22), which attract type 2 helper cells and regulatory T cells that express CCR4. CAR-T cells that are engineered to express CCR4 may have improved trafficking to the tumor site. **c**. Anti-CD30 CAR-T cells have been found to express PD-1, which suggests that they may be susceptible to the PD-1/PD-L1 pathway that leads to immune inhibition. In addition, Hodgkin Reed-Sternberg cells also express PD-L1, which may have an inhibitory effect on CAR-T cells expressing PD-1. Checkpoint inhibitors can interrupt the PD-1/PD-L1 pathway and lead to improved expansion and persistence of CAR-T cells. Growth factors, such as colony-stimulating factor 1 (CSF1) stimulate tumor associated macrophages (TAM) to be anti-inflammatory and promote tumor development. Combinations with CSF1 receptor (CSF1R) inhibitors could help interrupt the inhibitory tumor microenvironment and improve CAR-T cell efficacy

First of all, lymphodepleting regimens are to be thoroughly considered (Fig. 1a). Lymphodepleting or conditioning chemotherapy administered prior to CAR-T cell infusion clearly improve persistence and efficacy of CD19-directed CAR-T cells [33]. Lymphodepleting chemotherapy reduces the patient’s tumor burden and the number of suppressive cells [34–36]. The HL microenvironment, in particular, has numerous inhibitory cells including Tregs, T helper type 2 cells, and tumor-associated macrophages (TAM) [37, 38], which support the survival of Hodgkin Reed Sternberg (HRS) cells, the malignant cells in HL [39, 40]. Therefore, in HL, lymphodepletion may additionally make lymphoma cells more susceptible to CAR-T cell elimination by disrupting this inhibitory microenvironment. Finally, lymphodepletion removes competing sink cells, making IL-7 and IL-15 cytokines promptly available for CAR-T cell expansion [24, 36, 41].

The optimal lymphodepleting regimen to be used with CD30 CAR-T cells is not known. In their clinical trial of CD30-directed CAR-T cells, Wang et al. treated patients with 1 of 3 different conditioning regimens (consisting of fludarabine and cyclophosphamide, or gemcitabine, mustargen and cyclophosphamide, or nab-paclitaxel and cyclophosphamide), but did not find a statistically significant difference between them [31]. Many ongoing studies utilize fludarabine and cyclophosphamide as lymphodepletion, extrapolating from data with CD19-directed CAR-T clinical trials [42] (see Table 2; NCT02259556, NCT02917083, NCT03049449). An alternative regimen explored combines fludarabine and bendamustin as lymphodepletion (NCT02690545). Another approach is to infuse patients with CD30 CAR-T cells as consolidation after ASCT. In this scenario, ASCT acts as the ultimate lymphodepletion regimen, leading to high levels of stimulatory cytokines such as IL-7 and IL-15 that can support CAR-T cell expansion and eliminating suppressive lymphoid cells [43]. There is an ongoing clinical trial of CD30-directed CAR-T cells as consolidation after ASCT in patients with CD30+ lymphomas (NCT02663297).

Another important aspect to consider for CD30 malignancies is disease localization (Fig. 1b). Lymphomas are mainly a disease of lymphoid tissues (lymph nodes and bone marrow), but CD30+ tumors present further challenges. In HL, the chemokine environment is very important in influencing which cells accumulate in the tumor [44]. HRS cells produce thymus and activation-regulated chemokine/CC chemokine ligand 17 (TARC/CCL17) and macrophage-derived chemokine (MDC/CCL22). These chemokines attract cells that express their cognate receptor, CCR4, such as type 2 helper cells, Tregs, and myeloid derived suppressor cells (MDSC) [45–47]. The infiltration of these cells protects HRS cells by creating, not only a suppressive environment, but also a physical barrier from access by cytotoxic T lymphocytes. To ensure preferential trafficking to HL cells, Savoldo et al. created T cells that, in addition to expressing the CD30 CAR, also co-expressed the chemokine receptor, CCR4 [44]. They found that CD30-directed CAR-T cells that expressed CCR4 had improved migration to the tumor and increased anti-lymphoma activity compared to CD30-directed CAR-T cells that did not express CCR4 in HL mouse models [44]. One concern about this approach is that TARC and MDC are produced by other tissues, such as skin, which could increase toxicity. However, since CD30 is not expressed at these sites, the CD30-directed CAR-T cells should not cause on target off tumor toxicity. Instead, CD30-directed CAR-T cells that co-express CCR4 could also be more effective in CD30+ cutaneous lymphomas due to enhanced trafficking to skin. A clinical trial of CD30-directed CAR-T cells co-expressing CCR4 in patients with relapsed/refractory CD30+ lymphomas is planned to open in the near future.

As described above, the tumor environment of lymphomas and HL, in particular, is rich in inhibitory cells and molecules. Therefore, it is imperative to consider associating CAR CD30 with other immune regulators. Among candidate strategies, immune checkpoint inhibitors (ICIs, Fig. 1c) are key. This is especially interesting in HL, where ICIs have shown excellent single agent activity [48, 49]. In addition, Ramos et al. found that PD1 was expressed by 33% of infused CD30-directed CAR-T cells [32], which suggests that these cells will remain susceptible to the PD1/PDL1 inhibitory pathway once at the tumor site. In case reports of patients who progressed after receiving CD19-directed CAR-T cells and subsequently were treated with pembrolizumab, re-expansion of CD19 CAR-T cells and clinical response was observed [50, 51]. However, the optimal timing and sequencing of combination for ICIs and CD30 CAR-T cells needs to be identified. In addition, the effect of this combination on immune related adverse events and cytokine release syndrome is unknown. Alternatively, with advances in gene engineering, selective downregulation of inhibitory receptors by CAR T cells represent intriguing alternatives [24].

The presence of MDSC and their role in tumor protection in HL also calls for testing of combinations with novel modulators such as colony-stimulating factor 1 receptor (CSF1R) inhibitors, since MDSCs express CSF1R [52]. In addition, growth factors, such as CSF1, stimulate tumor associated macrophages to be anti-inflammatory, or the M2 phenotype, and promote tumor growth [52]. Increased number of tumor associated macrophages is associated with worse prognosis in HL [47]. This further supports the rationale of CSF1R inhibitors in HL and phase I studies on CSF1R inhibitors have been tested in HL and show good tolerance but limited efficacy [53]. However, combinations with CD30CAR T cells may prove beneficial.

## Conclusions

CAR-T cells have emerged as one of the most exciting new therapies for patients with hematologic malignancies, including lymphoma. CD30 is a promising new target to study given its universal expression in HL and ALCL and expression in a percentage of other types of lymphoma, and generally minimal risk of off tumor on target toxicity. Pre-clinical studies have further proved the feasibility of CD30-directed CAR-T cells. Clinical trials to date in patients with CD30+ lymphoma have shown that CD30-directed CAR-T cells are safe and have demonstrated some activity in patients with heavily treated relapsed and refractory disease. Possible modifications to further enhance activity of CD30-directed CAR-T cells include: 1) the identification of the ideal lymphodepleting regimen, 2) improvement in migration of CAR-T cells to the tumor site, 3) combination with novel therapies, such as checkpoint inhibitors or further engineering. There are several ongoing and upcoming clinical trials investigating CD30-directed CAR-T cells with different constructs, lymphodepletion regimens, and further modifications and we expect this therapy to be further developed and optimized over the coming years.

## Abbreviations

ALCL: Anaplastic large cell lymphoma
ALK+: Anaplastic lymphoma kinase-positive
alloSCT: Allogeneic stem cell transplant
ASCT: Autologous stem cell transplant
BV: Brentuximab vedotin
CAR: Chimeric antigen receptor
CAR-T: Chimeric antigen receptor T cells
CCR4: CC chemokine receptor 4
CD: Cluster of differentiation
CR: Complete response
CSF1R: Colony-stimulating factor 1 receptor
DLBCL: Diffuse large B-cell lymphoma
EBV: Epstein Barr Virus
EBV-CTLs: Epstein Barr Virus-specific cytotoxic T cells
FDA: Food and Drug Administration
HL: Hodgkin lymphoma
HRS: Hodgkin Reed Sternberg
HSPCs: Hematopoietic stem and progenitor cells
ICIs: Immune checkpoint inhibitors
MDC/CCL22: Macrophage-derived chemokine/CC chemokine ligand 22
MDSC: Myeloid-derived suppressor cells
ORR: Overall response rate
PD1: Programmed cell death protein 1
PDL1: Programmed death-ligand 1
PR: Partial response
scFv: Single chain variable fragment
SD: Stable disease
TAM: Tumor-associated macrophages
TARC/CCL17: Thymus and activation-regulated chemokine/CC chemokine ligand 17
TNF: Tumor necrosis factor
Tregs: Regulatory T cells
